# Autocatalytic, Brain Tumor‐Targeting Delivery of Bardoxolone Methyl Self‐Assembled Nanoparticles for Glioblastoma Treatment

**DOI:** 10.1002/smsc.202400081

**Published:** 2024-05-22

**Authors:** Zhang Ye, Wendy C. Sheu, Huan Qu, Bin Peng, Jia Liu, Li Zhang, Fanen Yuan, Yuxin Wei, Jiangbing Zhou, Qianxue Chen, Xuan Xiao, Shenqi Zhang

**Affiliations:** ^1^ Department of Neurosurgery Renmin Hospital of Wuhan University Wuhan Hubei 430060 China; ^2^ Department of Biomedical Engineering Yale University New Haven CT 06510 USA; ^3^ Department of Neurosurgery Yale University New Haven CT 06510 USA; ^4^ Department of Neurosurgery Pittsburgh University Pittsburgh PA 15260 USA; ^5^ Department of Ophthalmology Laboratory Medicine Center Renmin Hospital of Wuhan University Wuhan Hubei 430060 China

**Keywords:** bardoxolone methyl, glioblastoma, p28 peptide, self‐assembled nanoparticles

## Abstract

Glioblastoma multiforme (GBM) is a formidable cancer to treat due to the lack of effective drugs that can also efficiently cross the blood–brain barrier (BBB). Herein, a novel strategy involving the synthesis of p28 peptide‐conjugated, lexiscan (LEX)‐loaded, bardoxolone methyl (BM) self‐assembled nanoparticles, designated as p28‐LBM NPs, is introduced. These NPs are designed to overcome the dual challenges of effectively killing GBM cells and efficiently penetrating the brain. The p28 peptide is chosen for targeted delivery to brain tumors, and LEX is employed to enhance drug penetration across the BBB. The successful penetration of brain tumors by the p28‐LBM NPs after intravenous administration is demonstrated, with BM delivered as part of the NPs significantly inhibiting GBM tumor growth and extending the survival of mice with tumors. In conclusion, the p28‐LBM NPs present a promising approach for GBM treatment, with potential for effective and safe clinical applications in the future.

## Graphical abstract

Herein, a novel nanotechnology‐based approach for glioblastoma treatment is developed and validated. Autocatalytic, brain tumor‐targeting nanoparticles composed of self‐assembled bardoxolone methyl are synthesized. The resulting p28‐LBM nanoparticles show remarkable capability in penetrating brain tumors and demonstrate high efficacy in inhibiting glioblastoma growth. This approach can represent a promising new avenue for improving outcomes in patients with glioblastoma.

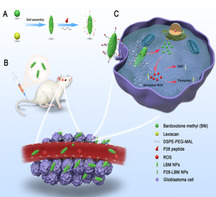

## 1 Introduction

Glioblastoma multiforme (GBM) is one of the most common and aggressive primary malignant brain tumors in adults. GBM is characterized by rapid growth, aggressiveness, and resistance to conventional therapies, contributing to a poor prognosis and a high recurrence rate.^[^ ^1^ ^]^ Currently, the standard of care for patient with GBM involved surgical resection, radiotherapy, and chemotherapy, primarily using the alkylating agent temozolomide.^[^ ^2^ ^]^ However, this approach has shown limited improvement in patient survival, with a median survival time of only 14‐16 months postdiagnosis.^[^ ^3^ ^]^ Therefore, development of novel therapies with improved efficacy is greatly needed.

For treatment of GBM, drug delivery remains a main obstacle, due to the existence of the blood–brain barrier (BBB). The BBB is a unique feature of the cerebral vasculature that blocks the entry of a large number of substances, including most drugs, into the brain, thereby impeding the treatment of brain tumors.^[^ ^4^, ^5^ ^]^ Therefore, improved treatment of GBM requires efficiently overcoming the BBB. In our previous studies, we showed that lexiscan (LEX), an adenosine A1A receptor agonist, can be incorporated into nanoparticles (NPs) to enhance BBB permeability,^[^ ^4^, ^6^ ^]^ and further enhancing the delivery of NPs to tumor tissues through an autocatalytic effect.^[^ ^6^ ^]^

Recently, we discovered a group of phytochemicals, which exist in medicinal plants and are capable of self‐assembly into NPs. We showed that NPs derived from selected phytochemicals could be employed as both a therapeutic agent and a drug deliver carrier for treatment of various diseases.^[^ ^7^, ^8^, ^9^, ^10^ ^]^ In this study, we identified bardoxolone methyl (BM), a new phytochemical that can form NPs through self‐assembly. While previous studies focused on the application of BM for ischemic diseases and diabetes,^[^ ^11^, ^12^, ^13^, ^14^ ^]^ we found that BM in NP form is highly effective in killing GBM cells through inhibition of epithelial–mesenchymal transition (EMT) and induction of pyroptosis. To improve penetration into brain tumors, we engineered BM NPs through surface conjugation of p28 and internal encapsulation of LEX. We showed that the resulting p28 peptide‐conjugated, LEX‐loaded, BM NPs, or p28‐LBM NPs, efficiently accumulated in tumors in the brain after intravenous administration, leading to effective treatment of GBM.

## 2 Results

### 2.1 Experiment Design

**Figure 1:**
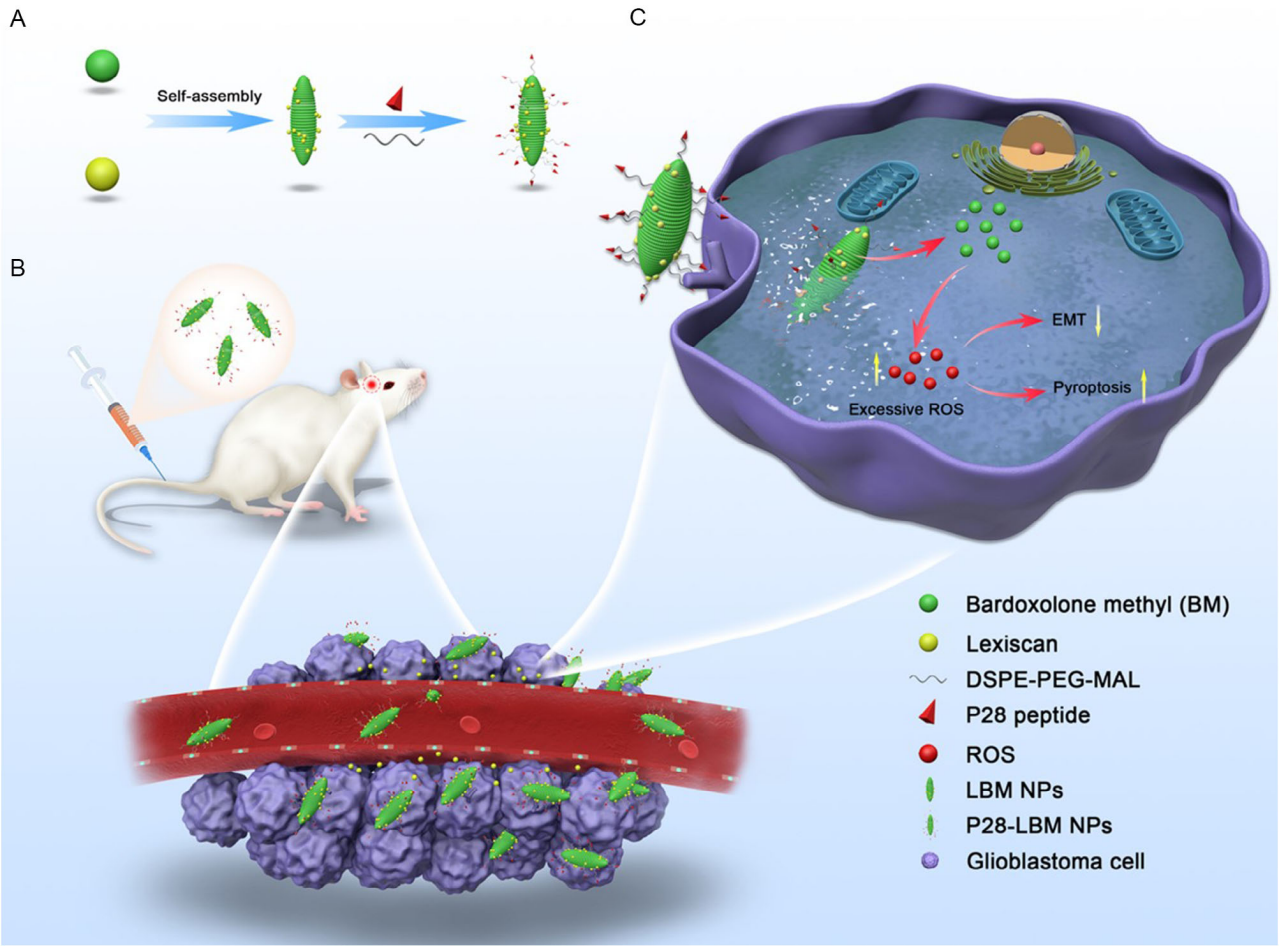
A) Schematic diagram of p28‐LBM NPs synthesis. B) Application of p28‐LBM NPs delivery to GBM. C) The mechanism of inhibiting GBM cells by p28‐LBM NPs.

To address the challenges associated with GBM drug delivery, we developed a nanotechnology approach to integrate tumor targeting and BBB penetration by synthesizing the multifunctional p28‐LBM NPs (**Figure** **1**A). Specifically, to achieve tumor targeting, we surface‐conjugated p28 peptide onto the self‐assembled BM NPs through linker DSPE‐PEG‐MAL. To address BBB penetration, LEX was encapsulated in the p28‐LBM NPs to modulate the BBB. After reaching the tumor microenvironment, p28‐LBM NPs release LEX locally, transiently enhance BBB permeability, and thereby allowing more NPs to penetrate the BBB (Figure 1B). The process improved delivery efficiency through autocatalysis. After reaching tumor microenvironment, the p28‐LBM NPs effectively inhibited GBM development through induction of pyroptosis and inhibition of EMT via production of excess reactive oxygen species (ROS) (Figure 1C).

### 2.2 Synthesis and Characterization of p28‐BM NPs

**Figure 2:**
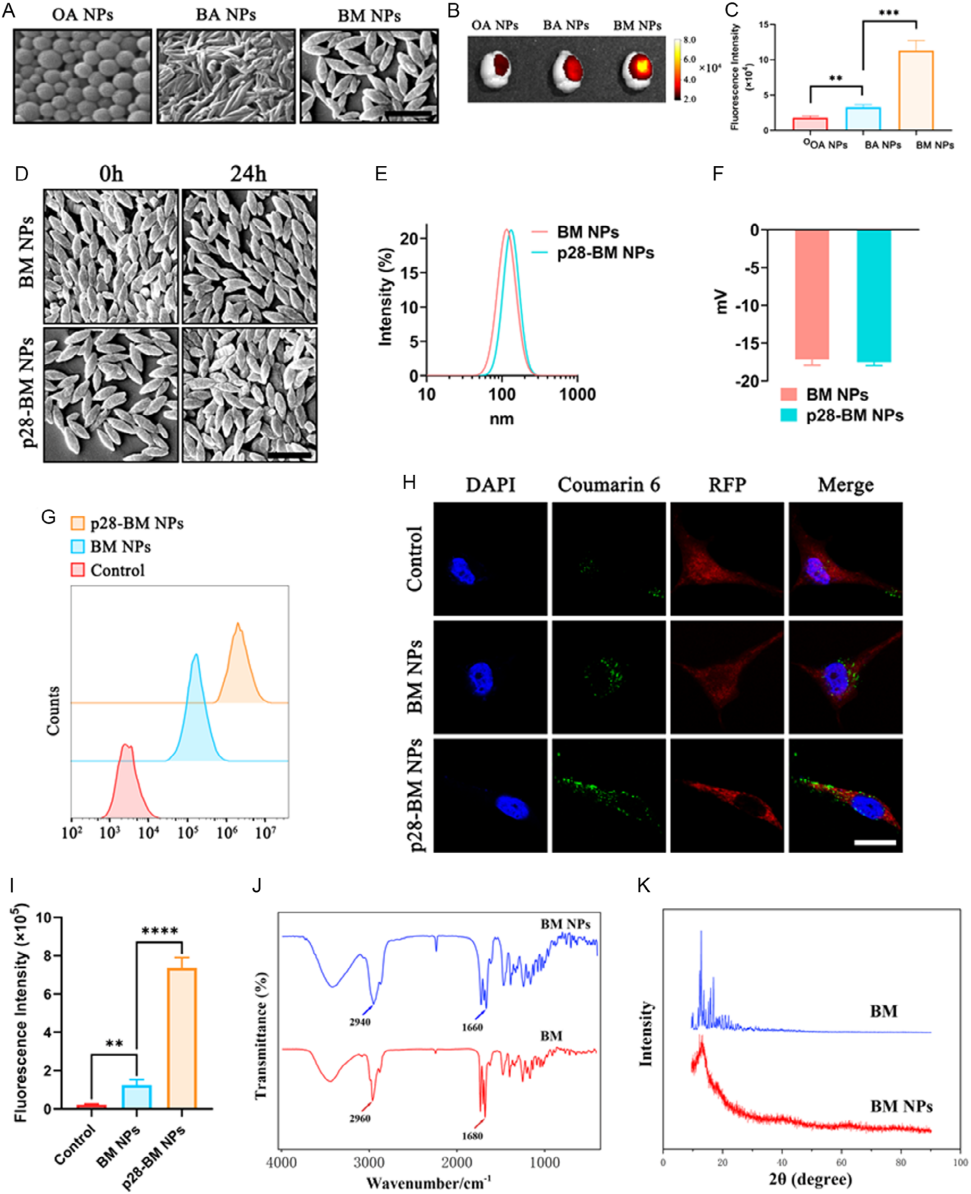
Synthesis and characterization of NPs. A) Synthesis of OA, BA, BM NPs of comparable size. Scale bar: 200 nm. Fluorescence intensity of B) NPs in isolated brain tissues and C) their semiquantitative analysis. D) SEM analysis of NP morphology. Scale bar: 200 nm. E) Hydrodynamic diameter of NPs. F) Zeta‐potential of NPs. G) Flow cytometry analysis of U87 cells incubated with free C6, BM NPs encapsulated with C6, and p28‐BM NPs encapsulated with C6 for 3 h. H) Fluorescence images and I) intensity quantification of U87 cells incubated with free C6, C6‐encapsulated BM NPs, and C6‐encapsulated p28‐BM NPs for 3 h. Scale bar: 20 μm. J) FTIR analysis revealed that the C—H and C=O groups were blueshifted after BM self‐assembled to form BM NPs. K) XRD analysis indicated that BM exists in BM NPs in an amorphous state. Statistical differences were determined by two‐tailed student's *t*‐test (*n* = 3; **P < 0.01, ***P < 0.001, ****P < 0.0001).

We previously showed that oleanolic acid (OA) and betulinic acid (BA), two phytochemicals, were capable of self‐assembling into NPs through emulsion.^[^ ^8^, ^9^, ^15^ ^]^ Following the same procedures, we found that BM NPs formed spindle‐shaped NPs, with a diameter of 50‐80 nm and a length of ≈170 nm (**Figure** **2**A). We characterized BM NPs for drug delivery to brain tumors in mice bearing U87 GBM tumors. Control mice were treated with OA NPs, which were spherical in shape with a diameter of ≈126 nm, and BA NPs, which are rod‐shaped with a diameter of 60‐80 nm and a length of ≈400 nm. All NPs were synthesized with encapsulation of IR780, an infrared dye allowing for noninvasive imaging through the in vivo imaging system (IVIS). NPs were normalized to ensure that each mouse received the same amount of fluorescence. We found that, compared to OA NPs and BA NPs, BM NPs had a significantly greater ability to target tumors in the brain (Figure 2B,C).

To further improve the delivery efficiency, we engineered BM NPs through surface conjugation of p28. p28 is a peptide derived from azurin,^[^ ^16^ ^]^ and has the ability to selectively penetrate human cancer cells, exerting cytostatic and cytotoxic effects while protecting normal cells.^[^ ^17^ ^]^ It has been previously shown that p28 peptide can be used to enhance delivery of imaging agents and NPs to tumors.^[^ ^18^, ^19^, ^20^ ^]^ We found that surface conjugation of p28 peptide did not affect the morphology and size of BM NPs (Figure 2D,E). The zeta potential of BM NPs was measured to be −17.4 ± 0.7 mV, similar to that of p28‐BM NPs, which was determined to be −17.6 ± 0.3 mV (Figure 2F). We evaluated the potential of glioma cell lines to internalize p28‐BM NP by incorporating a fluorescent dye, C6, into the synthetic NPs to allow for noninvasive imaging. Upon incubating the NPs with U87 cells for an hour, we quantified cellular uptake utilizing fluorescence microscopy and flow cytometry. Flow cytometric analysis indicated a notably higher internalization of p28‐BM NPs, shown by the increased fluorescence intensity of C6 in the cells, compared to BM NPs at the 3 h mark, supporting our preliminary hypothesis that the surface‐conjugation of p28 can enhance the cellular uptake of the NPs (Figure 2G). Fluorescence microscopy further validated our findings, revealing the extensive dispersion of p28‐BM NPs throughout the cytoplasm (Figure 2H,I).

Fourier‐transform infrared spectroscopy (FTIR) analysis showed that peaks corresponding to the stretching vibrations of C—H and C═O groups in free BM were present at 2960 and 1680 cm^−1^, respectively (Figure 2J). Upon the assembly of BM into BM NPs, these peaks underwent a blueshift to 2940 and 1660 cm^−1^, respectively, implying the establishment of intermolecular hydrogen bonds. This evidence underlined the formation of BA NPs as a process driven by intermolecular hydrogen bonding.

X‐Ray powder diffraction (XRD) analysis offered further substantiation of BM NPs self‐assembly (Figure 2K). The XRD pattern of free BM displayed sharp characteristic peaks at 2*θ* values of 12.87°, 13.33°, and 17.40°, indicative of a high degree of crystallinity. In contrast, the XRD patterns of BM NPs lacked sharp characteristic peaks, suggesting an amorphous state of BM within BM NPs.

### 2.3 Surface‐Conjugation of p28 on BM NPs Allows Targeted Delivery to GBM

**Figure 3:**
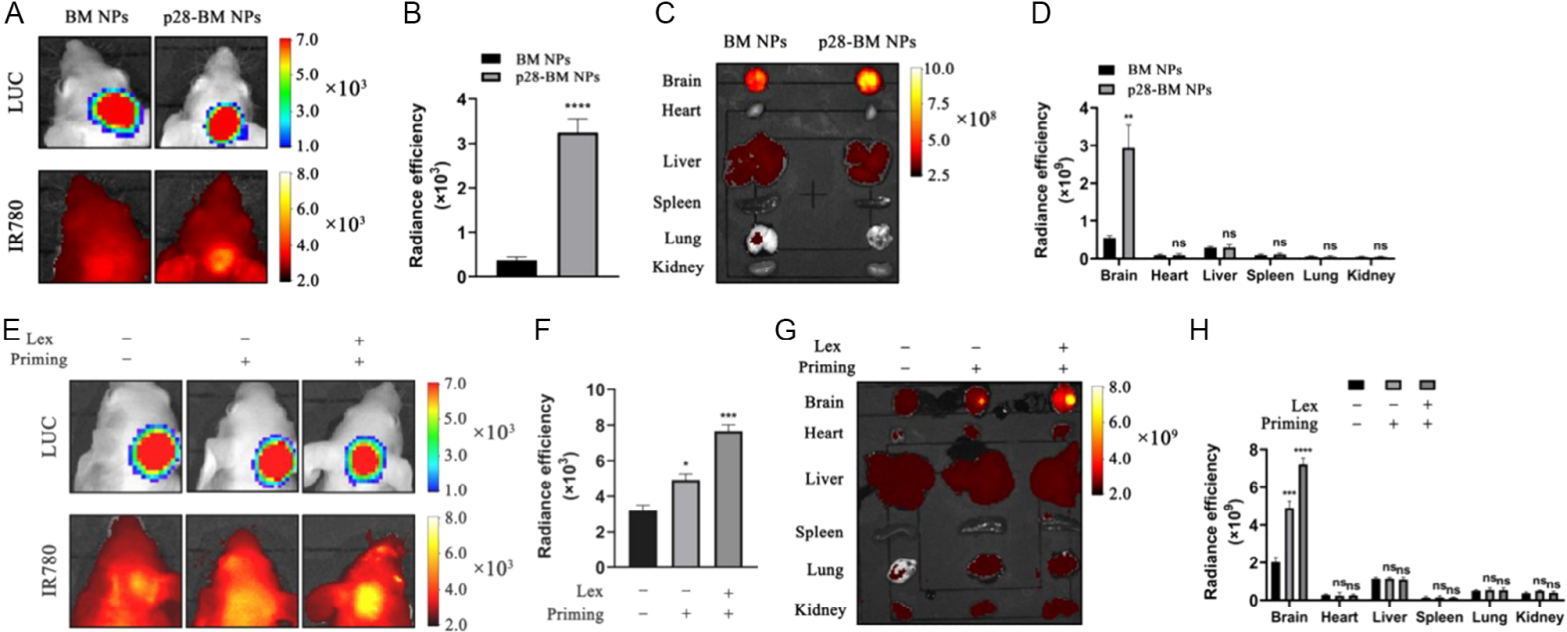
Targeted delivery of NPs to the GBM. A) Representative image and B) semiquantification of NPs in U87 GBM‐bearing mice receiving the indicated treatments. C) Representative images and D) semiquantification of NPs in the heart, liver, spleen, lung, and kidney of U87 GBM‐bearing mice receiving the indicated treatments. E) Representative images and F) semiquantification of NPs in U87 GBM‐bearing mice receiving the indicated treatments. G) Representative images and H) semiquantification of NPs in the heart, liver, spleen, lung, and kidney of U87 GBM‐bearing mice receiving the indicated treatments. Statistical differences were determined by two‐tailed student's *t*‐test (*n* = 3; ns, not significant, *P < 0.05, **P < 0.01, ***P < 0.001, ****P < 0.0001).

We assessed if conjugation of p28 enhanced delivery of NPs to tumors. p28 is an azurin derivative peptide which preferentially penetrated various cancer cells and could be potentially employed as ligand for cancer‐targeting drug delivery.^[^ ^16^, ^17^, ^20^, ^21^ ^]^ NPs with and without the p28 peptide were synthesized with encapsulation of IR780 and administered to mice bearing GBM tumors derived from U87 cells that were engineered to express luciferase. Mice were selected based on luciferase imaging to ensure that all bear comparable tumor burdens. Imaging based on IR780 showed a significant augmentation in NPs concentration within tumors upon p28 conjugation (**Figure** **3**A,B). Further analysis of isolated organs using indicated that p28 significantly enhanced NP accumulation within tumors, with a substantial 5.3‐fold increase, while avoiding significant buildup within peripheral organs (Figure 3C,D).

### 2.4 LEX Enhances Intracranial Drug Delivery of p28‐BM NPs Through Autocatalysis

We previously showed that LEX could modulate on the permeability of the BBB and encapsulation of LEX‐enhanced delivery of NPs to tumors through an autocatalytic mechanism.^[^ ^6^, ^22^, ^23^, ^24^ ^]^ We determined if encapsulation of LEX could further accumulation of p28‐BM NPs in U87 tumors. LEX‐encapsulated, p28 peptide‐conjugated self‐assembled BM NPs were synthesized. The resulting p28‐LBM NPs contained LEX 0.55% by weight. We evaluated if LEX encapsulation could enhance the BBB penetration in an engineering BBB model, in which hCMEC/D3 cells were grown on top of the insert membrane while astrocyte normal human astrocyte (NHA) cells grown on the bottom (Figure S1, Supporting Information). Partially BBB disruption was induced by treatment with CoCl_2_ and verified through measurement of transepithelial/transendothelial electrical resistance (TEER). We found that, compared to control groups, p28‐LBM NPs exhibited the greatest permeability to cross the BBB. Then BBB penetration capabilities of NPs were further evaluated in mice bearing U87 GBM tumor. After priming with NPs twice, the mice received intravenous administration of NPs with encapsulation of IR780 at day 3. Control mice were treated with IR780‐loaded NPs without priming. After 24 h, the mice were imaged. Our results revealed that delivery without LEX priming did not significantly enhance the delivery efficiency. However, when delivered with LEX priming, the efficiency was amplified by 3.5‐fold, confirming that the autocatalytic mechanism further enhances drug delivery capacity (Figure 3E,F). IVIS imaging and quantification of fluorescence intensity in isolated organs showed that the p28‐LBM NPs were not significantly enriched in the heart, liver, spleen, lung, and kidney (Figure 3G,H). Characterization of p28‐LBM NPs demonstrated that morphology, size, and zeta potential were not significantly altered with the encapsulation LEX (Figure S2, Supporting Information).

### 2.5 p28‐LBM NPs Inhibit the Proliferation of GBM Cells

**Figure 4:**
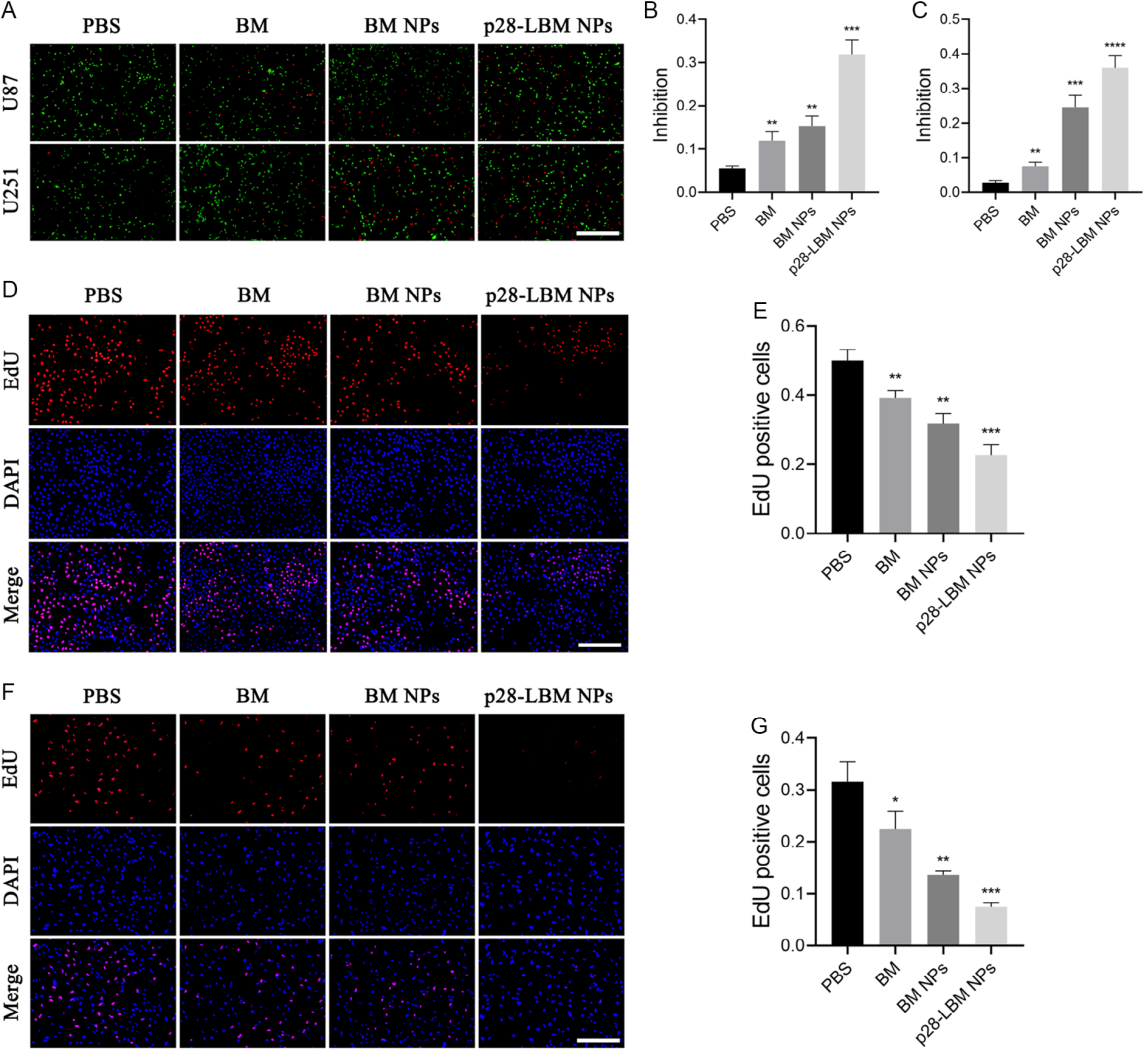
p28‐LBM NPs inhibit GBM cell proliferation. A) Representative images and semiquantification of the live/dead stating of cell viability assay in B) U87 and C) U251 cells receiving the indicated treatments. Scale bar: 200 μm. D) Representative images and E) semiquantification of EdU assay in U87 cells receiving the indicated treatments. Scale bar: 200 μm. F) Representative images and G) semiquantification of EdU assay in U251 cells receiving the indicated treatments. Scale bar: 200 μm. Statistical differences were determined by two‐tailed student's *t*‐test (*n* = 3; *P < 0.05, **P < 0.01, ***P < 0.001, ****P < 0.0001).

To evaluate the cytotoxicity of p28‐LBM NPs in GBM, we performed cell viability assays within two GBM cell lines, U87 and U251. Specifically, two fluorescent dyes, Calcein‐AM, and PI, are used to specifically label live/dead cells after treating the cells with the indicated treatments. Calcein‐AM emits an intense green fluorescence upon catalytic hydrolysis within the live cells, and PI stains dead cells with red fluorescence. The results showed that p28‐LBM NPs significantly induced cell death in both U87 and U251 cell lines (BM equivalents were 150 ng mL^−1^) (**Figure** **4**A). Quantitative analysis indicated an increased inhibition of proliferation in both U87 cells (Figure 4B) and U251 cells (Figure 4C) when treated with p28‐LBM NPs. The inhibitory effect of p28‐LBM NPs was substantial, with rates exceeding 30% in both tested cell lines. To further delineate the impact of p28‐LBM NPs on GBM cells, we employed an EdU assay to detect proliferating cells stained with red fluorescence. This analysis demonstrated that p28‐LBM NPs suppressed the proliferation of U87 (Figure 4D,E) and U251 (Figure 4F,G) cell lines more potently than both free BM and BM NPs. Together, these findings provide compelling in vitro evidence of the inhibitory efficacy of p28‐LBM NPs on GBM cell viability, pointing to their potential therapeutic application.

### 2.6 p28‐LBM NPs Induce Pyroptosis of GBM Cells

**Figure 5:**
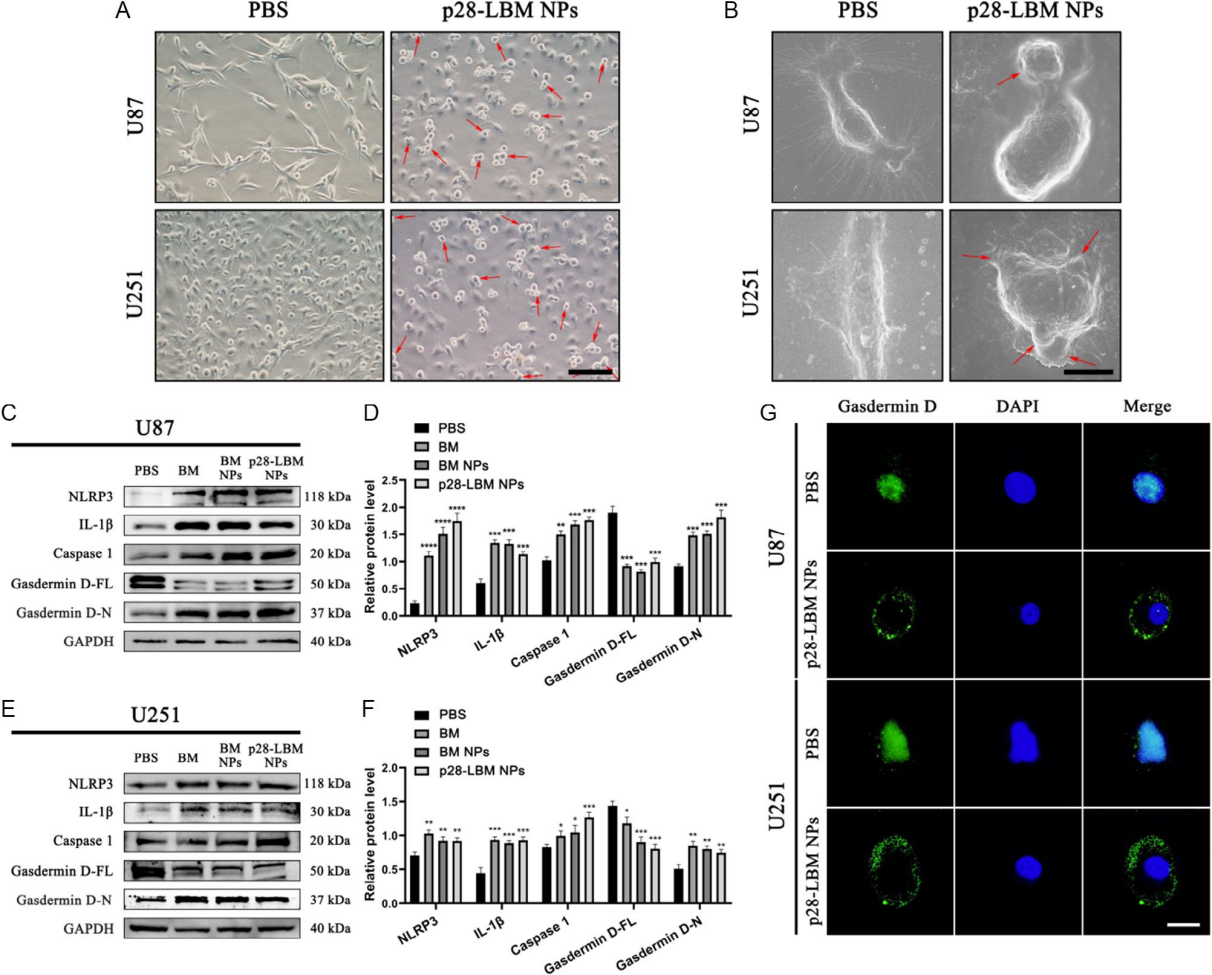
P28‐LBM NPs induce pyroptosis in GBM cells. A) Representative microscopy images of U87 and U251 cells with indicated treatments. Scale bar: 200 μm. B) Representative SEM images of U87 and U251 cells with indicated treatments. Scale bar: 20 μm. C) Western blot analysis and D) semiquantitative analysis of pyroptosis‐related markers in U87 cells after receiving the indicated treatments. E) Western blot analysis and F) semiquantitative analysis of pyroptosis‐associated markers in U251 cells after receiving the indicated treatment. G) Representative fluorescence microscopy images of GSDMD localization in U87 and U251 cells with indicated treatments. Scale bar: 10 μm. Statistical differences were determined by two‐tailed student's *t*‐test (*n* = 3; *P < 0.05, **P < 0.01, ***P < 0.001, ****P < 0.0001). Exposure time: 5 s.

Microscopic examination of p28‐LBM NP‐treated U87 and U251 cells revealed characteristic of pyroptosis cell morphology, including balloon‐like protrusions of the plasma membrane, suggesting the induction of GBM cell pyroptosis by p28‐LBM NPs (**Figure** **5**A,B). Pyroptosis represents a specialized form of programmed cell death characterized by gasdermin family‐mediated cytosolic pore formation, subsequent cell swelling, plasma membrane rupture, and massive release of proinflammatory cytokines, such as IL‐1β.^[^ ^25^ ^]^ This process unfolds through two primary pathways: 1) activation of caspase 1, which cleaves gasdermin D (GSDMD) into N‐ and C‐terminal fragments leading to N‐terminal GSDMD‐composed membrane pore formation, or 2) activation of caspase 3, resulting in a similar cleavage of gasdermin E and subsequent membrane pore formation by N‐terminal gasdermin E oligomers.^[^ ^26^ ^]^ Both classical pathways result in balloon‐like cell swelling and release of inflammatory factors.^[^ ^27^ ^]^ Common pyroptosis‐related markers are highly expressed in GBM, including NLRP3, caspase 1, and GSDMD(Figure S3A‐D, Supporting Information). A semiquantitative analysis of pyroptosis‐associated protein alterations in two GBM cell lines treated with phosphate‐buffered saline (PBS), free BM, BM NPs, and p28‐LBM NPs unveiled a significant increase in inflammatory vesicle NLRP3 and upregulation of activated caspase 1, most pronouncedly in the p28‐LBM NP‐treated group in both U87 (Figure 5C,D) and U251 (Figure 5E,F) cells. Additionally, activated N‐terminal GSDMD was upregulated, leading to a subsequent increase in IL‐1β and a corresponding reduction in full‐length GSDMD. Immunofluorescence staining further revealed a shift in the localization of GSDMD within both U87 and U251 cells treated with p28‐LBM NPs from the nucleus to the cytosol, alluding to the formation of an N‐terminal GSDMD‐composed membrane pore complex (Figure 5G). Collectively, our findings demonstrate that p28‐LBM NPs induce programmed cell death in GBM cells by triggering pyroptosis via the NLRP3/caspase 1/GSDMD signaling pathway, underscoring their potential therapeutic utility.

### 2.7 p28‐LBM NPs Inhibit the Migration and Invasion of GBM Cells

**Figure 6:**
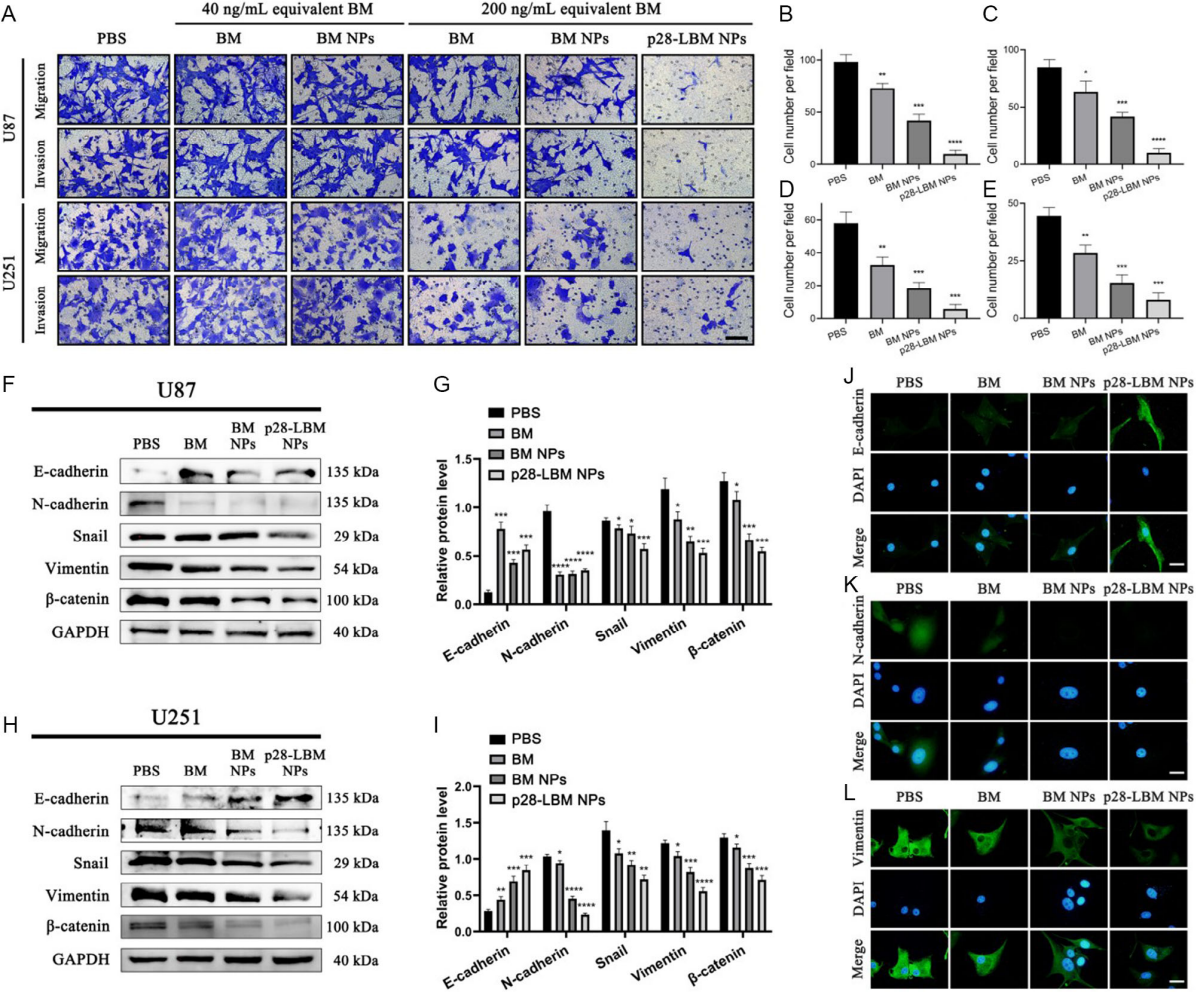
p28‐LBM NPs inhibit EMT in GBM cells. A) Representative images of transwell assays after receiving the indicated treatments in U87 and U251 cells. Quantitative analysis in U87 cell B) migration and C) invasion. Quantitative analysis in U251 cell D) migration and E) invasion. Scale bars: 100 μm. F) Western blot analysis and G) semiquantitative analysis of EMT‐related markers in U87 cells after receiving the indicated treatments. H) Western blot analysis and I) semiquantitative analysis of EMT‐related markers in U251 cells. Representative fluorescence microscopy images of J) E‐cadherin, K) N‐cadherin, and L) vimentin expression in U87 cells with indicated treatment. Scale bar: 20 μm. Statistical differences were determined by two‐tailed student's *t*‐test (*n* = 3; *P < 0.05, **P < 0.01, ***P < 0.001, ****P < 0.0001). Exposure time: 5 s.

We evaluated the effect of NP treatment on EMT, which is relevant to the malignancy of GBM and typically involves cell migration and invasion processes.^[^ ^28^ ^]^ We found that four common EMT‐related markers, E‐cadherin, N‐cadherin, snail, vimentin, and β‐catenin, were highly expressed in GBM (Figure S4, Supporting Information). We employed a transwell assay to evaluate the effect of p28‐LBM NPs (BM equivalent of 200 ng mL^−1^) on EMT (**Figure** **6**A) and found that p28‐LBM NPs exert a substantial inhibitory effect on EMT process in U87 (Figure 6B,C) and U251 cell lines (Figure 6D,E). Further analysis showed that p28‐LBM NPs modulated key molecular markers associated with EMT, as evidenced by the diminished expression of N‐cadherin, snail, vimentin, and β‐catenin, and the enhanced expression of E‐cadherin, as identified by western blotting in U87 (Figure 6F,G) and U251 cells (Figure 6H,I). Complementary to these findings, immunofluorescence staining of U87 cells verified an elevation in E‐cadherin (Figure 6J), along with reduction in the expression of N‐cadherin (Figure 6K) and vimentin (Figure 6L). Collectively, these data compellingly demonstrate that p28‐LBM NPs hinder the migration and invasion of GBM cells by specifically targeting and suppressing the EMT process through modulation of the β‐catenin signaling pathway.

To validate the effects of p28‐LBM NP treatment on pyroptosis and EMT, we determined the changes of key proteins associated with the programmed cell death pathway using proteomic sequencing. Consistently, the results showed a significant upregulation of proteins linked to the pyroptosis pathway and a significant downregulation of protein‐related EMT pathways in the treated group (Figure S5, Supporting Information).

### 2.8 p28‐LBM NPs Induce Pyroptosis in GBM Cells Through the Excessive Production of ROS

**Figure 7:**
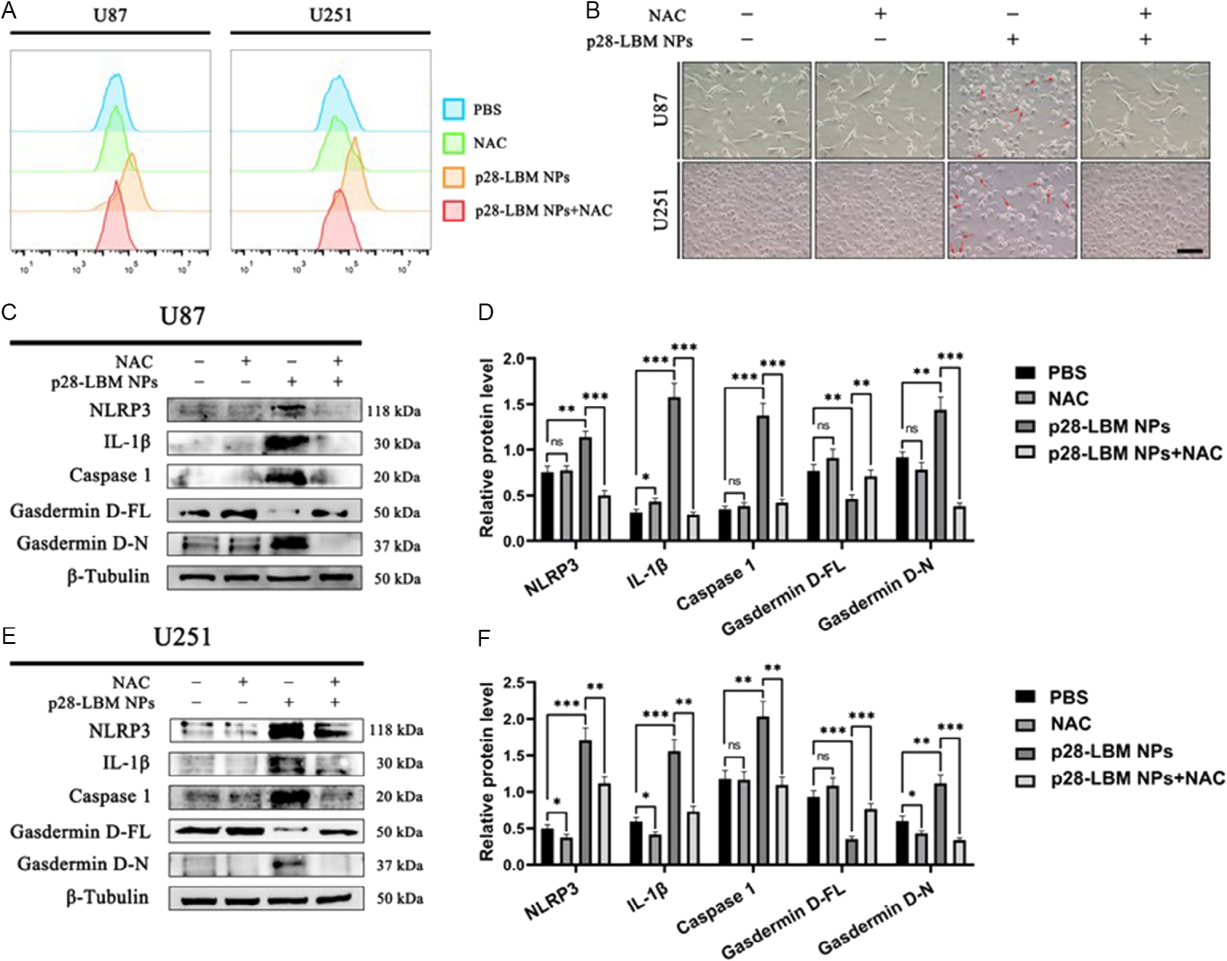
p28‐LBM NPs induce pyroptosis in GBM cells via excess ROS production. A) Flow cytometry analysis of ROS content in U87 and U251 cells after receiving the indicated treatments. B) Representative microscopy images of GBM cells after receiving the indicated treatments. Scale bar: 100 μm. C) Western blot analysis and D) semiquantitative analysis of pyroptosis‐related proteins in U87 cells after receiving the indicated treatments. E) Western blot analysis and F) semiquantitative analysis of pyroptosis‐associated proteins in U251 cells after receiving the indicated treatments. Statistical differences were determined by two‐tailed student's *t*‐test (*n* = 3; ns, not significant, *P < 0.05, **P < 0.01, ***P < 0.001, ****P < 0.0001). Exposure time: 5 s.

We determined if p28‐LBM NPs induce pyroptosis in GBM cells through the excessive production of ROS. Flow cytometry analysis of the ROS levels in the untreated U87 and U251 cells indicated no discernible ROS alterations when ROS inhibitor NAC was added (**Figure** **7**A). In contrast, a significant increase in ROS levels was detected in p28‐LBM NPs‐treated cells, and this increase was reversed by the addition of NAC^[^ ^29^ ^]^. Microscopic observation supported this finding, revealing that p28‐LBM NP induced pyroptosis, which was reversed when NAC was added (Figure 7B). Western blot analysis further substantiated our finding in both U87 (Figure 7C,D) and U251 cells (Figure 7E,F). In both GBM cell lines, NAC attenuated the formation of the inflammatory vesicle NLRP3, resulting in a subsequent reduction in the activation of caspase 1 and GSDMD, and ultimately leading to a decrease in the inflammatory factor IL‐1β. Overall, our study demonstrated that p28‐LBM NPs induced excessive ROS production, leading to cell pyroptosis.

### 2.9 p28‐LBM NPs Inhibit EMT in GBM cells Through the Excessive Production of ROS

**Figure 8:**
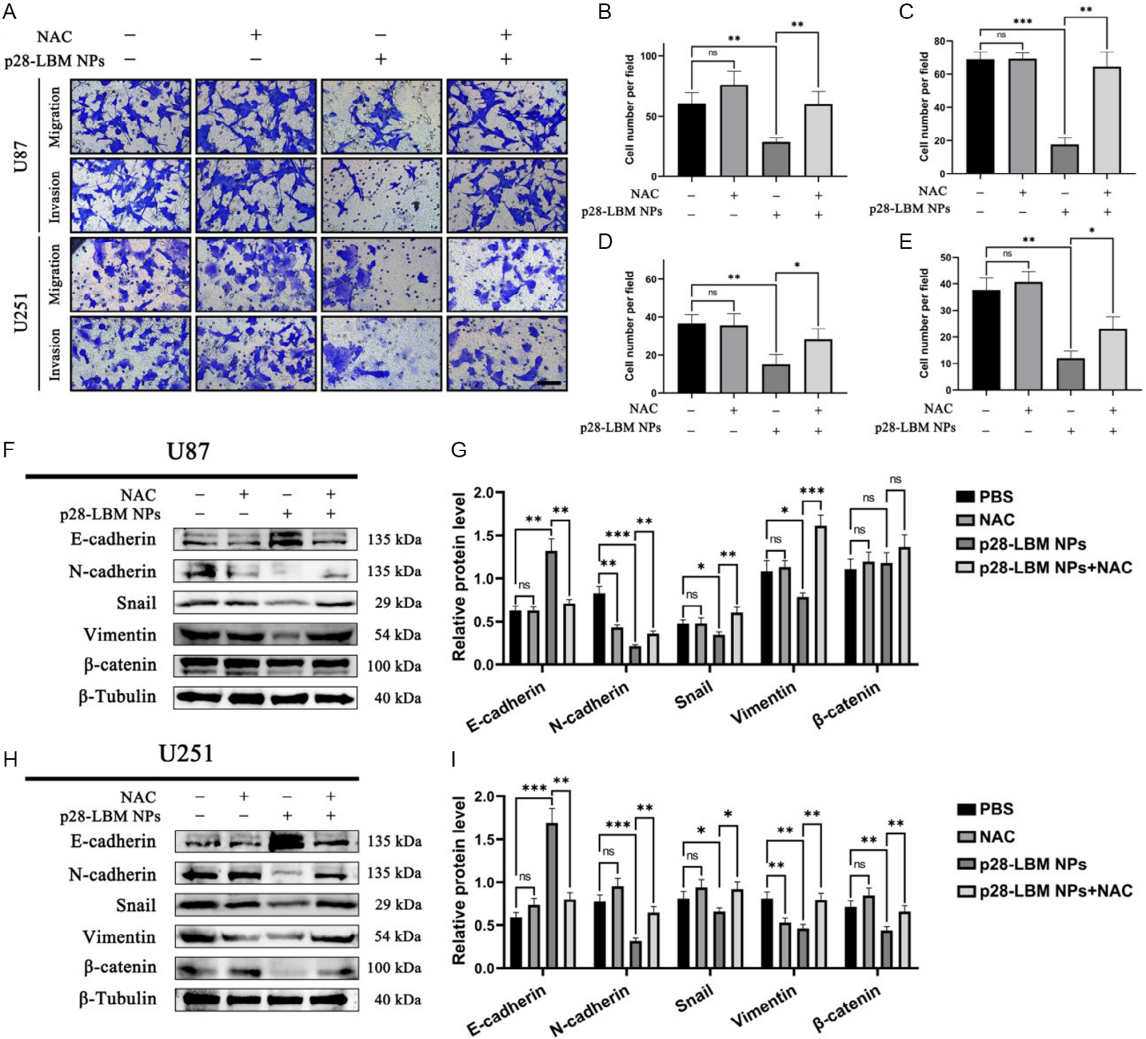
p28‐LBM NPs inhibit EMT in GBM cells through excess production of ROS. A) Representative images of transwell assays after receiving the indicated treatments in U87 and U251 cells. Quantitative analysis in U87 cell B) migration and C) invasion. Quantitative analysis in U251 cell D) migration and E) invasion. Scale bar: 100 μm. F) Western blot analysis and G) semiquantitative analysis of EMT‐related proteins in U87 cells after receiving the indicated treatments. H) Western blot analysis and I) semiquantitative analysis of EMT‐related proteins in U251 cells after receiving the indicated treatments. Statistical differences were determined by two‐tailed student's *t*‐test (*n* = 3; ns, not significant, *P < 0.05, **P < 0.01, ***P < 0.001, ****P < 0.0001). Exposure time: 5 s.

We determined if p28‐LBM NPs inhibit EMT in GBM cells also through excess ROS production. U87 and U251 cells were induced to produce excess ROS by treatment with p28‐LBM NPs, followed by treatment with ROS inhibitor NAC. Changes in the EMT phenotype were analyzed via a transwell assay. Microscopic imaging indicated that p28‐LBM NPs decreased migration and invasion in both GBM cell lines (**Figure** **8**A). Quantitative analysis further demonstrated that excess ROS production by p28‐LBM NPs leads to such inhibition in migration and invasion in U87 cells (Figure 8B,C) and U251 cells (Figure 8D,E), as evidenced by the addition of NAC. Western blot analysis of EMT‐associated proteins corroborated our initial conjecture. Specifically, NAC reduced E‐cadherin expression while simultaneously restoring N‐cadherin, snail, and vimentin expression in both U87 cells (Figure 8F,G) and U251 cells (Figure 8H,I).

### 2.10 p28‐LBM NPs Suppress GBM Progression in vivo

**Figure 9:**
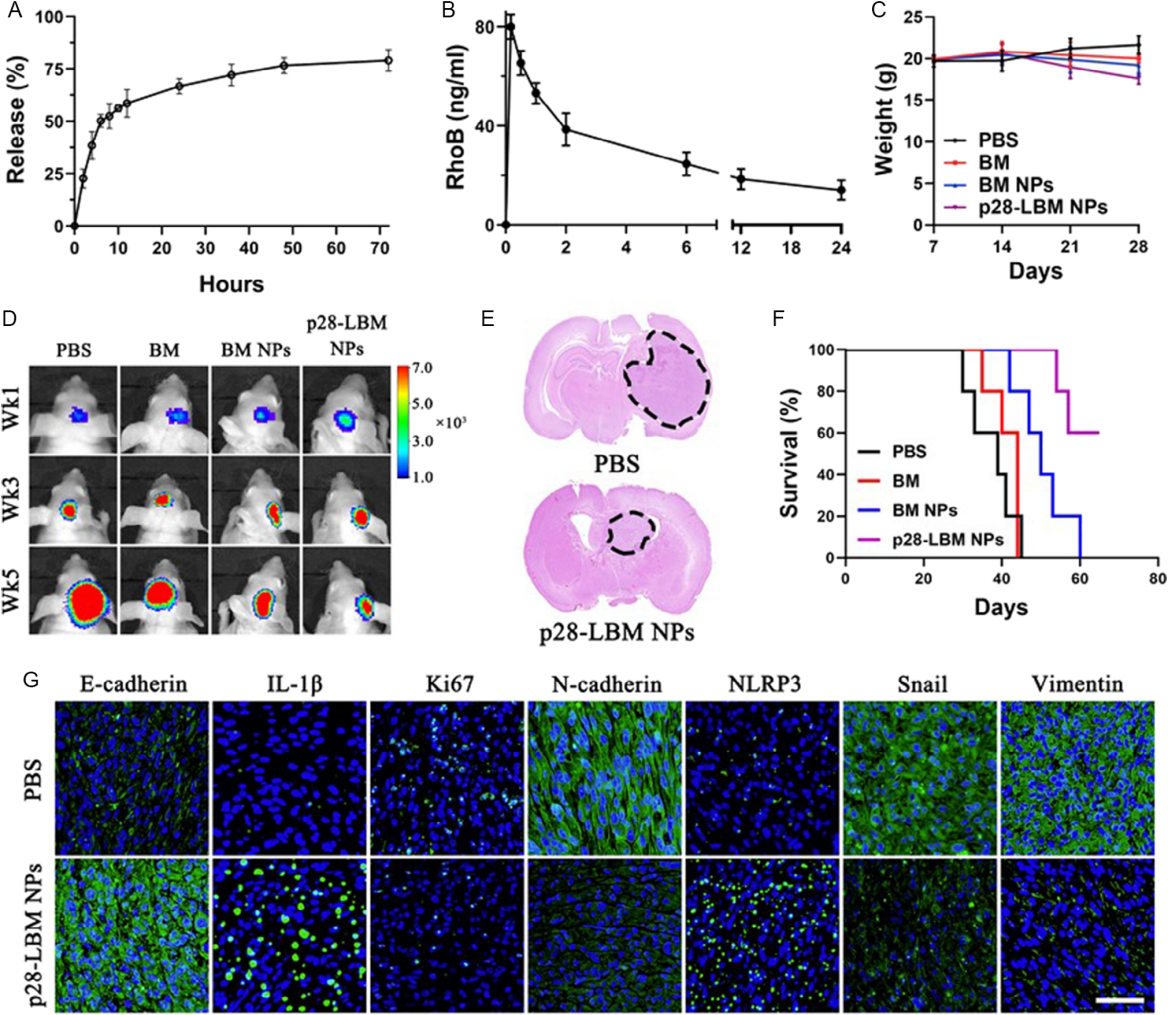
Systemic treatment of p28‐LBM NPs suppresses GBM progression in vivo. A) Controlled release of p28‐LBM NPs in PBS. B) Plasma concentrations of RhoB against time in mice after intravenous injection of p28‐LBM NPs loaded with RhoB. C) Change in body weight of U87 GBM‐bearing mice receiving the indicated treatments over time. D) Representative images of luciferase signals in IVIS imaging of mice that received the indicated treatments. E) H&E staining of brains isolated from mice receiving the indicated treatments. F) Kaplan–Meier survival curves of U87 GBM‐bearing mice receiving the indicated treatments (*n* = 5). G) Immunofluorescence staining (E‐cadherin, IL‐1β, Ki67, N‐cadherin, NLRP3, snail, vimentin) of brains isolated from mice that received the indicated treatments.

We explored the systemic delivery of p28‐LBM NPs to the brain for treatment of GBM. In PBS, p28‐LBM NPs released over 50% of BM within 6 h and 75% within 72 h (**Figure** **9**A). The pharmacokinetics of p28‐LBM NPs were evaluated in mice using Rhodamine B (RhoB)‐loaded p28‐LBM NPs. Following tail vein administration, blood samples were periodically collected, and RhoB concentrations were plotted over time (Figure 9B). The in vivo half‐life of p28‐LBM NPs in the circulatory system was determined to be 15.07 h. Notably, no significant weight loss was observed in the mice, indicating minimal systemic toxicity, and suggesting potential safety for intravenous administration (Figure 9C). Analyses of serum alanine aminotransferase and aspartate aminotransferase assays, as well as hematoxylin and eosin (H&E) staining of major organs also did not identify obvious liver toxicity or tissue damage, respectively (Figure S6, Supporting Information). To further assess the therapeutic efficacy of p28‐LBM NPs, luciferase‐expressed U87 tumor‐bearing mice were treated intravenously with a dose equivalent to 2 mg kg^−1^ of BM. The dose and treatment duration for mice were determined based on previously published literature on BM.^[^ ^30^, ^31^ ^]^ Controls included mice treated with PBS, free BM, or BM NPs. After 4 weeks of thrice‐weekly treatments, we observed a pronounced suppression of tumor growth in the p28‐LBM NP group, more so than the free BM and BM NPs cohorts (Figure 9D). Histological analyses after 5 weeks of treatment revealed reduced tumor sizes and malignancy in the p28‐LBM NP‐treated group compared to the PBS cohort (Figure 9E). Kaplan–Meier survival analysis revealed that p28‐LBM NP treatment significantly improved survival in GBM‐bearing mice (*P* < 0.01). Median survival times for the groups were as follows: p28‐LBM NPs (59 days), PBS (39 days), free BM (44 days), and BM NPs (52 days) (Figure 9F). Furthermore, immunofluorescence staining of EMT‐ and pyroptosis‐related markers highlighted suppressed EMT and enhanced pyroptosis in the tumors of the p28‐LBM NP‐treated group (Figure 9G). In conclusion, our findings underscore the efficacy of p28‐LBM NPs in impeding GBM progression, potentially by suppressing EMT and activating pyroptosis. Further, a peptide dose equivalent of 200 ng mL^−1^ did not induce detectable cytotoxicity in U87 cells (Figure S7, Supporting Information), suggesting that the p28 peptide present on the NP surface is not responsible for the observed antitumor effects.

## 3 Discussion

Advancements in GBM treatment necessitate the development of innovative therapeutic strategies. In our prior research, we screened a range of phytochemical NPs for treatment of stroke and breast cancer.^[^ ^9^, ^23^, ^32^, ^33^ ^]^ However, the potential of phytochemical nanomaterials for GBM treatment has not been explored. In this study, we identified BM as a phytochemical nanomaterial that could be also used for effective treatment of GBM. We demonstrated that BM formed spindle‐shaped NPs. Compared to spherical NPs, spindle‐shaped NPs have a higher aspect ratio (length to width ratio).^[^ ^34^, ^35^, ^36^ ^]^ There is evidence that elongated shape could facilitate the interaction with cell membrane, thereby leading to enhanced cellular uptake.^[^ ^37^, ^38^ ^]^ The spindle shape might also allow for improved diffusion and penetration through the tortuous spaces within tumors compared to spherical particles,^[^ ^39^ ^]^ thereby enabling better delivery. Consistently, we found that BM NPs accumulated in tumors with efficiency significantly greater than spherical OA NPs (Figure 2B,C). We showed that the delivery efficiency of BM NPs could be further improved through surface conjugation of p28 peptide and internal encapsulation of LEX (Figure 3). We showed that the resulting p28‐LBM NPs effectively killed GBM cells through induction of pyroptosis by activating the NLRP3/caspase 1/GSDMD signaling pathway. This, in turn, leads to the release of inflammatory factors and changes in cellular morphology.^[^ ^40^ ^]^ Additionally, the p28‐LBM NPs have the ability to inhibit the migration and invasion of GBM cells by targeting the EMT process through modulation of the β‐catenin signaling pathway.^[^ ^41^ ^]^ We further characterized p28‐LBM NPs in vivo and demonstrated that intravenous administration of p28‐LBM NPs effectively inhibited the progression of GBM development and extended the survival of tumor‐bearing mice.

## 4 Conclusion

We discovered BM as a novel phytochemical nanomaterial capable of forming spindle‐shaped NPs. We showed that, likely due to their unique physical properties, BM NPs efficiently penetrated GBM in the brain, and the efficiency could be further enhanced through a p28‐ and LEX‐mediated tumor‐targeting autocatalytic mechanism. We demonstrated the resulting p28‐LBM NPs as a therapeutic agent for effective treatment of GBM. Given their remarkable ability to penetrate brain tumors and high efficacy in inhibiting GBM, p28‐LBM NPs may represent a promising avenue for improving the treatment of GBM patients.

## 5 Experimental Section

5.1

5.1.1

##### Materials

BM was obtained from TOPSCIENCE. The following polyclonal antibodies were purchased from Proteintech: Glyceraldehyde 3‐phosphate dehydrogenase (GAPDH), E‐cadherin, N‐cadherin, snail, vimentin, β‐catenin, NLRP3, IL‐1β, caspase 1, and GSDMD. The p28 peptide with the sequence LSTAADMQGVVTDGMASGLDKDYLKPDDC was acquired from GenScript.

##### Synthesis of BM NPs, p28‐BM NPs, and p28‐LBM NPs

BM NPs and p28‐BM NPs were synthesized using standard emulsion procedures. The procedure for encapsulating hydrophobic cargoes (IR780, coumarin 6) within p28‐BM NPs was as follows: A mixture of 1 mg of the indicated hydrophobic cargo, 10 mg of BM, and 1 mg of DSPE‐PEG2000‐Mal was dissolved in a solution of 2 mL dichloromethane (DCM) and 0.1 mL methanol. Additionally, 0.25 mg LEX was added in this step for p28‐LBM NPs. This mixture was added dropwise to 5 mL of 2.5% polyvinyl alcohol (PVA) solution. The resulting mixture was sonicated at 0 °C for 120 s (5 s on, 3 s off) and then added to 40 mL of 0.3% PVA solution. The NP solution was obtained after overnight evaporation of DCM under magnetic stirring. To acquire BM/LBM NPs with surface display of maleimide moieties, the solution was centrifuged at 18 000 rpm for 30 min.

The p28 peptide contains a cysteine with a thiol group (SH) at its terminus. To conjugate the p28 peptide to BM NPs, maleimide–thiol click chemistry was employed. Specifically, an aqueous solution of p28 peptide (3 mg) was mixed with tris(2‐carboxyethyl)phosphine (0.3 mg) and incubated for 1 h. The resulting solution was then added to an aqueous solution of BM NPs (30 mg) and magnetically stirred overnight at 4 °C (520 rpm). After completion, the solution was centrifuged at 18 000 rpm for 30 min to obtain p28‐BM NPs/p28‐LBM NPs. The resulting NPs were found to contain LEX at 0.55% by weight.

The quantity of p28 conjugated to the DSPE‐PEG2000‐Mal polymer was inferred by calculating the difference between the total peptide introduced in the reaction and the peptide amount found in the supernatants after the polymer precipitation steps. The calculation formula is as follows

(1) $$\text{Conjugation efficiency }\left(\%\right)=\frac{\text{Total p}28\text{ peptide}-\text{Recovered p}28\text{ peptide}}{\text{Total p}28\text{ peptide}}\times 100$$

##### Scanning Electron Microscopy

The morphology of the NPs was analyzed using scanning electron microscopy (SEM). To briefly summarize the process, a solution containing NPs at a concentration of 1 mg mL^−1^ was applied to a silicon wafer and allowed to air‐dry. Next, the sample was coated with a layer of gold using a sputterer at a current of 40 mA for 60 s. Subsequently, images were captured using a field emission scanning electron microscope (Zeiss GeminiSEM 500, Oberkochen, Germany) at an acceleration voltage of 5 kV.

##### Dynamic Light Scattering

The hydrodynamic size and zeta‐potential of the NPs were assessed using dynamic light scattering. In a nutshell, 1 mg of NPs was mixed with 10 mL of deionized water (ddH_2_O) and then measured using a Malvern Zetasizer (Zetasizer Nano ZSP, Malvern, UK).

##### Drug Loading and Release Study

To characterize drug loading, the NPs were dissolved in DMSO to release LEX, which was subsequently quantified using HPLC (Agilent 1100, Agilent, USA).

For drug release characterization, p28‐BM NPs were placed inside a dialysis bag with a molecular weight cutoff of 3000. The dialysis bag containing the NPs was then immersed in a 40 mL PBS solution under controlled conditions, placed at 37 °C, and shaken at 120 times per minute. At specific time intervals (ranging from 2 to 72 h), 1 mL of the solution was removed from outside the dialysis bag and replaced it with 1 mL of fresh PBS. Subsequently, the amount of BM released was determined using HPLC (Agilent 1100, Agilent, USA).

##### Bioinformatics Analysis

Gene expression data were collected from 172 GBM samples within The Cancer Genome Atlas database, omitting any data that lacked prognostic information. The data processing and the creation of the corresponding graphs were carried out using RStudio.

##### Cell Culture

Human GBM cell lines, U87 and U251, were obtained from the cell bank of the Shanghai Institute of Biochemistry and Cell Biology (Shanghai, China). The cells were cultured in Dulbecco's Modified Eagle's Medium (DMEM) (Servicebio, China), supplemented with 10% fetal bovine serum (Gibco, Australia), and maintained at 37 °C in a 5% CO_2_ environment.

##### Immunofluorescence Assays

The samples were fixed for 10 min and subsequently treated with 0.1% Triton‐X for 30 min. After that, the cells were blocked with 1% BSA for 1 h to prevent nonspecific binding. Following the blocking step, the cells were incubated with the primary antibody (Abclonal, China) overnight at 4 °C. The next day, the cells were exposed to the secondary antibody (Proteintech, China) for 1 h in the dark. To visualize the cell nuclei, 4′,6‐diamidino‐2‐phenylindole (DAPI) (Servicebio, China) was used for staining. Finally, the stained samples were examined, and images were captured using an Olympus BX53 microscope (Olympus).

##### Western Blotting

Cells were lysed in radio‐immunoprecipitation assay buffer for 30 min at 4 °C. A BCA kit (Beyotime, China) was used to detect the protein concentration. The proteins were then separated using 12.5% SDS‐PAGE, transferred to polyvinylidene fluoride membranes, and incubated with the primary antibody (Abclonal, China) overnight at 4 °C, followed by the secondary antibody (Servicebio, China) for 1 h. Visualization of the proteins was achieved with a ChemiDoc gel imaging system (Bio‐Rad, USA). The grayscale values of the blots were quantified using ImageJ software, and the target proteins were normalized against GAPDH or β‐Tubulin to determine relative protein levels.

##### Cell Viability Assay

The cells were subjected to predetermined treatment with BM equivalent of 150 ng mL^−1^. A 96‐well plate was seeded with 2000 cells per well, followed by the addition of 10 μL of CCK‐8 solution to each well. The plate was then incubated in a cell incubator for 1 h. After incubation, the absorbance of each well was measured using a microplate reader.

##### Cell Proliferation Assay

We detected cell proliferation using an EdU kit (Beyotime, China). Briefly, the cells were cultured in a medium containing 10 μM EdU for one day, and then treated with 4% paraformaldehyde for 30 min. Afterward, we permeabilized them with 0.5% Triton X‐100 for 30 min and incubated them with a 1× Apollo reaction cocktail for another 30 min. Following this, the cells were stained with DAPI for 10 min. Images were captured using an Olympus BX53 microscope.

##### Transwell Assay of Migration and Invasion

A transwell assay was used to evaluate the effect of p28‐LBM NPs (BM equivalent of 200 ng mL^−1^) on migration and invasion. The PBS‐treated group and the nontoxic concentration of BM (40 ng mL^−1^)‐treated group were used as controls for the transwell assay. U87 and U251 cells were treated with the indicated treatment for 24 h, placed in the Matrigel‐coated upper chamber for invasion (or uncoated for migration) with serum‐enriched medium in the lower chamber.

##### Measurement of ROS

2′,7′‐Dichlorofluorescein diacetate (H2DCF‐DA) (Sigma, USA) was utilized to detect intracellular ROS. Following predetermined treatments, cells were harvested and subsequently resuspended in PBS. They were then incubated with 20 μM H2DCF‐DA at 37 °C for 30 min in the dark. Finally, the fluorescence intensity of various groups was assessed using flow cytometry (CytoFlex, Beckman Coulter, USA).

##### Cellular Uptake

To further characterize the uptake of NPs by GBM cells, BM NPs and p28‐BM NPs were loaded with coumarin‐6 (C6). After incubating the C6‐NPs with U87 cells for 1 h, the cells were fixed in paraformaldehyde. Cellular uptake was investigated using an Olympus BX53 microscope (Olympus) and flow cytometry (CytoFlex, Beckman Coulter, USA).

##### Transwell BBB Model

The transwell BBB model was constructed following established protocols. Initially, transwell inserts underwent coating with poly‐l‐lysine at a concentration of 0.001%. Subsequently, NHA cells were placed on the basolateral aspect of these inserts and allowed to settle for 4 to 5 h before the insert was turned over. On the following day, hCMEC/D3 cells were introduced to the apical side. The model reached maturity once the TEER values hit 30 Ω^*^ cm^2^. For BBB permeability simulation, a 24 h exposure to 500 μM of CoCl_2_ was applied. Following this, the apical side received fluorescein isothiocyanate (FITC)‐tagged NPs, which were premixed with endothelial cell growth medium, while the opposite side was filled with 700 μL of complete DMEM. Monitoring of FITC levels was conducted at intervals ranging from 30 min to 72 h, using a microplate reader to collect data from the basolateral fluid samples.

##### Mass Spectrometric Analysis

U87 cells subjected to the indicated treatment were labeled with tandem mass tag (TMT) sixplex reagents using a TMT Mass Tagging Kit from Thermo Scientific, according to the manufacturer's instructions. These samples were then analyzed with an EASY‐nLC1000/LTQ Orbitrap Elite.

##### In vivo Studies

Our study was approved by the ethics committee of Wuhan University Renmin Hospital. BALB/c‐nu mice were bought from Hunan Slack Kingda Laboratory Animal Company. BALB/c‐nu mice were administered intravenously with RhoB‐loaded p28‐LBM NPs (*n* = 3), and blood was collected at predetermined time points following p28‐LBM NP injection. RhoB concentrations in the plasma were then quantified. For characterizing NPs in GBM treatment, tumor‐bearing mice were divided into four groups and treated with PBS, free BM, LBM NPs, or p28‐LBM NPs. IVIS imaging of intracranial tumors was conducted at 1, 3, and 5 weeks after tumor inoculation to assess tumor progression. Fourteenth day post‐tumor inoculation, treatment was administered 3 days per week for 4 weeks. Mice were monitored daily for weight changes and survival. One group (*n* = 3) of mice was euthanized 5 weeks postinoculation for pathological staining. The brains were harvested, fixed, sliced, and subsequently subjected to tissue immunofluorescence staining.

##### Statistical Analysis

All data were collected in triplicate unless stated otherwise. Data are presented as mean values ± standard deviations (SD). Comparisons between groups were analyzed using student's *t*‐test, performed with GraphPad Prism 8.0. Survival of animals in in vivo studies were analyzed based on Kaplan–Meier analyses. *P* < 0.05 (*), 0.01 (**), 0.001 (***), and 0.0001 (****) were considered significant.

## Conflict of Interest

The authors declare no conflict of interest.

## Supporting information

Supplementary Material

## Data Availability

The data that support the findings of this study are available from the corresponding author upon reasonable request.
