# Supplementary material for: Autocatalytic, Brain Tumor‐Targeting Delivery of Bardoxolone Methyl Self‐Assembled Nanoparticles for Glioblastoma Treatment
Source: Small Sci. 2024 May 22;4(8):2400081. doi: 10.1002/smsc.202400081 (PMC11935168; doi:10.1002/smsc.202400081)
Supplement: Supplementary file 1 — Supplementary Material [file SMSC-4-2400081-s001.pdf]

Supporting Information

Autocatalytic, brain tumor-targeting delivery of bardoxolone methyl self-assembled nanoparticles for glioblastoma treatment

Zhang Ye [1#], Wendy C. Sheu [2#], Huan Qu [1#], Bin Peng [3], Jia Liu [3], Li Zhang [1], Fanen Yuan [4], Yuxin Wei [1], Jiangbing Zhou [2,3], Qianxue Chen [1*], Xuan Xiao [5*], Shenqi Zhang [1*]

[1]Department of Neurosurgery, Renmin Hospital of Wuhan University, Wuhan, Hubei, China, 430060

[2]Department of Biomedical Engineering, Yale University, New Haven, CT, 06510, USA.

[3]Department of Neurosurgery, Yale University, New Haven, CT, 06510, USA.

[4]Department of Neurosurgery, Pittsburgh University, Pittsburgh, PA, 15260, USA.

[5]Department of Ophthalmology, Laboratory Medicine Center, Renmin Hospital of Wuhan University, Wuhan, Hubei, China, 430060

#These authors contributed equally to this work.

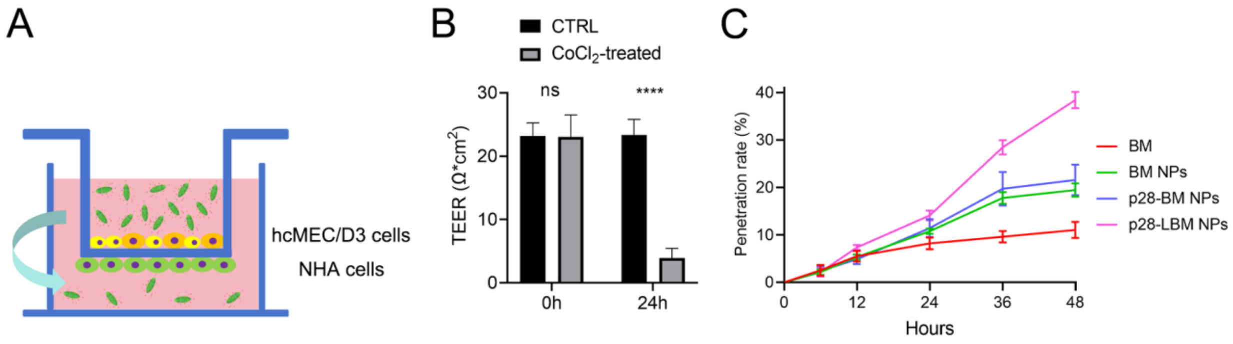

Figure S1. In vitro characterization of the ability of BM and BM NPs to cross the blood brain barrier (BBB). (A) Schematic diagram of the transwell-based BBB penetration assay. (B) TEER measurements in the group with and without CoCl2 Treatment. (C) Quantification of the ability of bardoxolone methyl and indicated NPs to cross the BBB. ****P<0.0001.

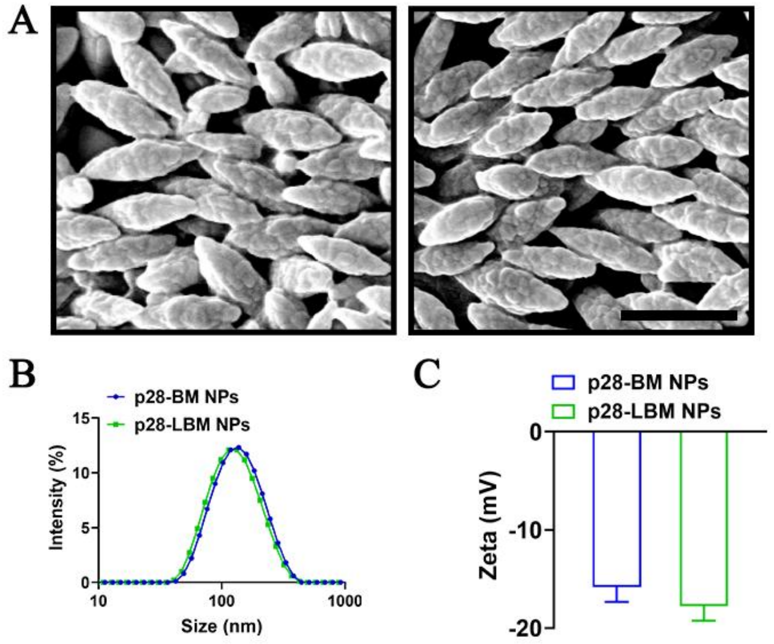

Figure S2. Characterization of p28-BM and p28-LBM NPs. (A) Representative SEM images of the indicated NPs. Scale bar: 150 nm. (B) DLS analysis of the hydrodynamic diameters of NPs. (C) Zeta-potential of NPs (n=3).

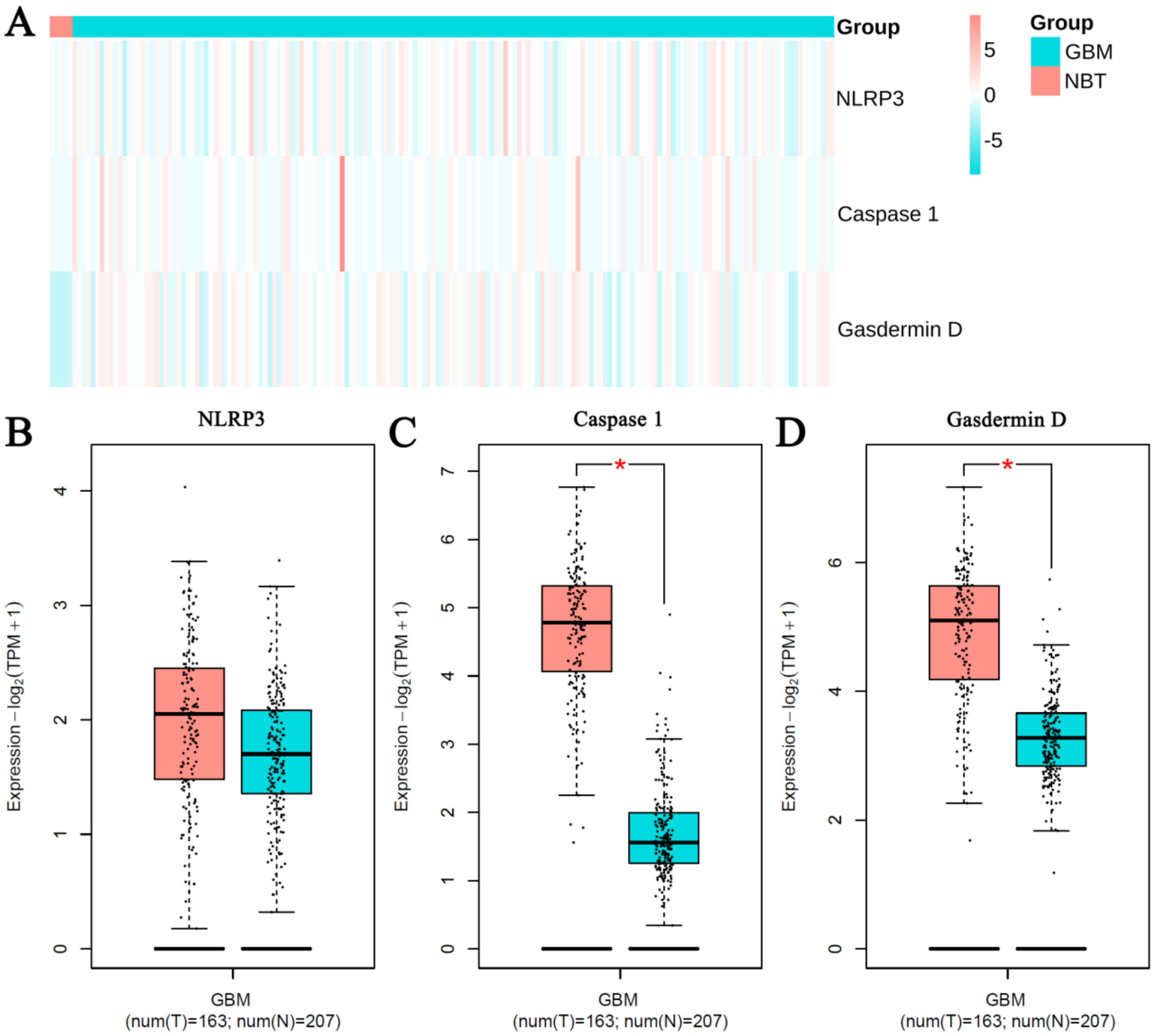

Figure S3. Common pyroptosis-related markers in GBM. Expression profiles (A) and differential analysis (B-D) of pyroptosis-related proteins in normal brain tissue and GBM tissue in the TCGA database and GTEx database. (*, $P < 0.05$).

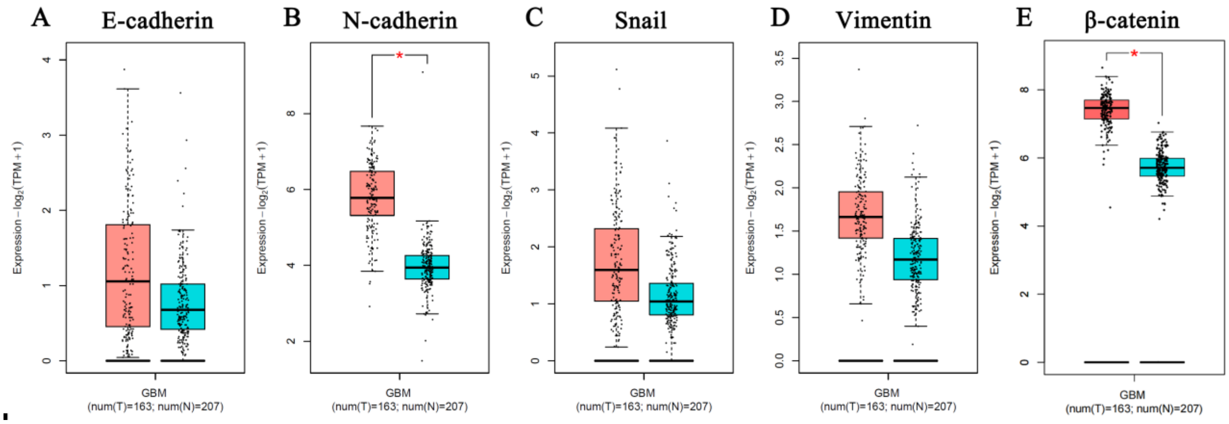

Figure S4. Common EMT-related proteins in GBM. (A-E) Differential analysis of EMT-related proteins in normal brain tissue and GBM tissue in the TCGA and GTEx database. (*, $P < 0.05$).

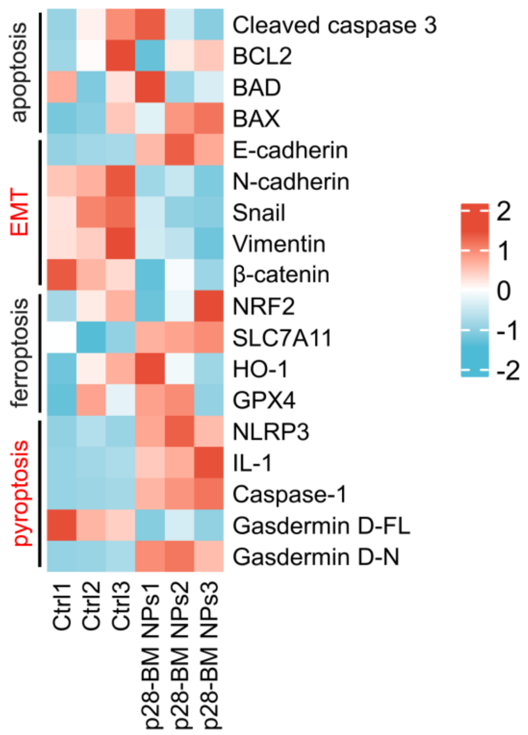

Figure S5. Mass spectrometric analysis of key proteins relevant to cellular apoptosis, EMT, ferroptosis and pyroptosis in control and p28-BM NPs-treated cells.

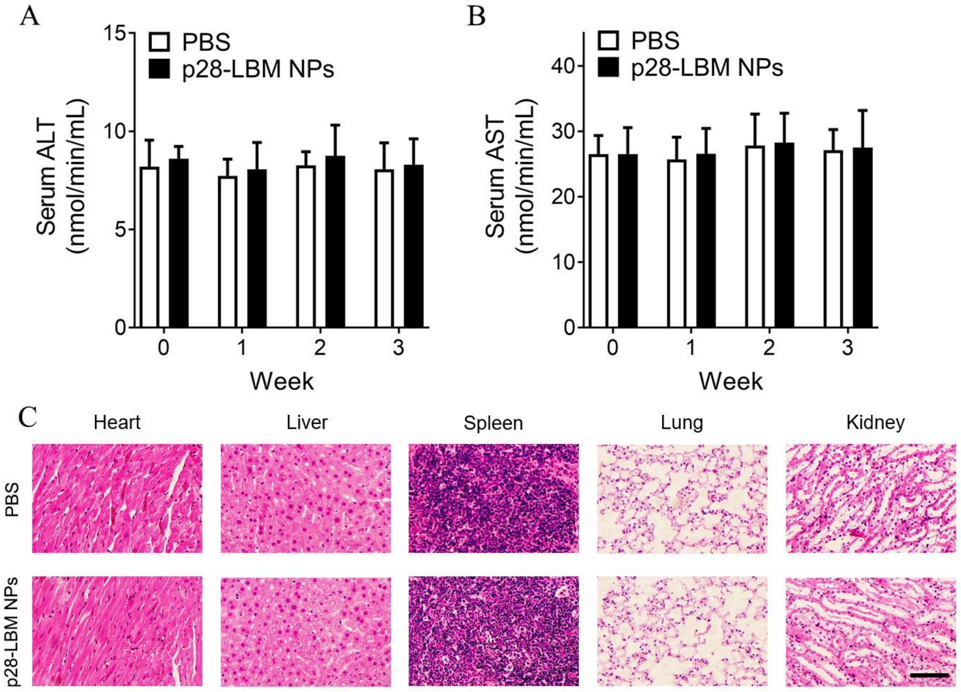

Figure S6. Characterization of the cytotoxicity of p28-LBM NPs. Quantification of serum ALT (A) and AST (B) levels in mice received the indicated treatments. (n=3). (C) Representative H&E staining images of major organs isolated from mice receiving the indicated treatments. Scale bar: 200 μm.

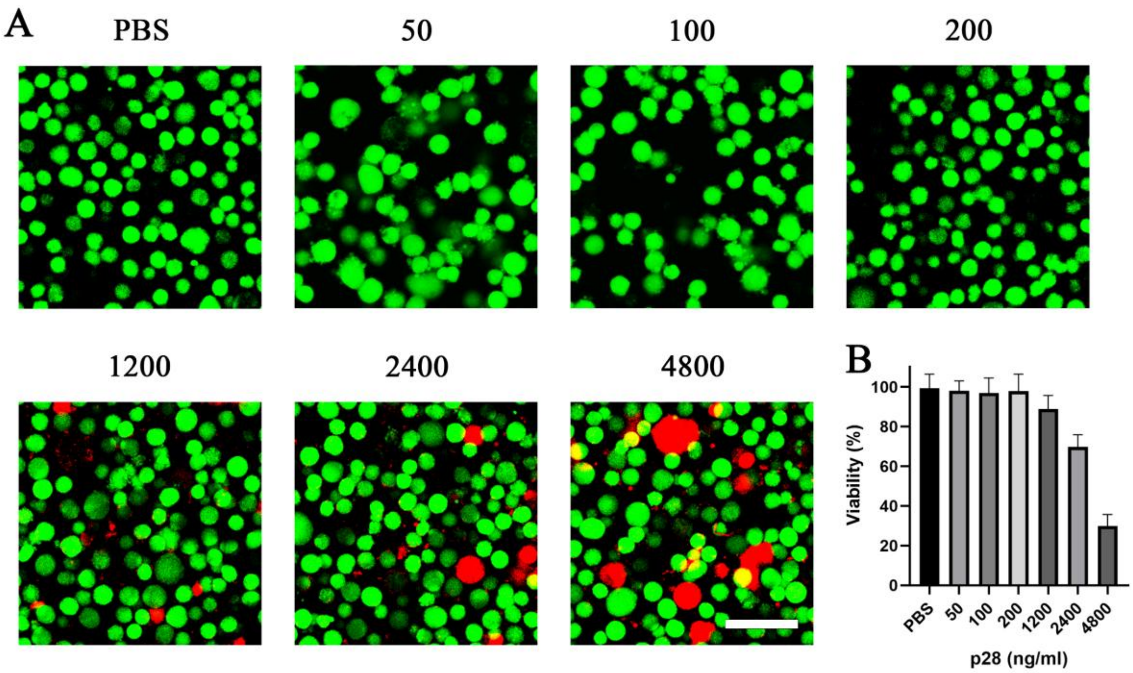

Figure S7. Characterization of the cytotoxicity of p28 peptide. (A) Live/dead staining of cell viability assay of U87 cells treated with p28 peptide at the indicated concentrations (ng/ml). Scale bar: 50 µm. (B) CCK-8 assay quantifying viability of U87 cells treated with p28 peptide at the indicated concentrations. (n=3).
